# Microparticles Mediate Hepatic Ischemia-Reperfusion Injury and Are the Targets of Diannexin (ASP8597)

**DOI:** 10.1371/journal.pone.0104376

**Published:** 2014-09-15

**Authors:** Narci C. Teoh, Hussam Ajamieh, Heng Jian Wong, Kevin Croft, Trevor Mori, Anthony C. Allison, Geoffrey C. Farrell

**Affiliations:** 1 Australian National University Medical School at The Canberra Hospital, Canberra, ACT, Australia; 2 School of Medicine and Pharmacology, University of Western Australia, Perth, WA, Australia; 3 Alavita Inc, Palo Alto, California, United States of America; National University of Singapore, Singapore

## Abstract

**Background & Aims:**

Ischemia–reperfusion injury (IRI) can cause hepatic failure after liver surgery or transplantation. IRI causes oxidative stress, which injures sinusoidal endothelial cells (SECs), leading to recruitment and activation of Kupffer cells, platelets and microcirculatory impairment. We investigated whether injured SECs and other cell types release microparticles during post-ischemic reperfusion, and whether such microparticles have pro-inflammatory, platelet-activating and pro-injurious effects that could contribute to IRI pathogenesis.

**Methods:**

C57BL6 mice underwent 60 min of partial hepatic ischemia followed by 15 min–24 hrs of reperfusion. We collected blood and liver samples, isolated circulating microparticles, and determined protein and lipid content. To establish mechanism for microparticle production, we subjected murine primary hepatocytes to hypoxia-reoxygenation. Because microparticles express everted phosphatidylserine residues that are the target of annexin V, we analyzed the effects of an annexin V-homodimer (Diannexin or ASP8597) on post-ischemia microparticle production and function.

**Results:**

Microparticles were detected in the circulation 15–30 min after post-ischemic reperfusion, and contained markers of SECs, platelets, natural killer T cells, and CD8^+^ cells; 4 hrs later, they contained markers of macrophages. Microparticles contained F2-isoprostanes, indicating oxidative damage to membrane lipids. Injection of mice with TNF-α increased microparticle formation, whereas Diannexin substantially reduced microparticle release and prevented IRI. Hypoxia-re-oxygenation generated microparticles from primary hepatocytes by processes that involved oxidative stress. Exposing cultured hepatocytes to preparations of microparticles isolated from the circulation during IRI caused injury involving mitochondrial membrane permeability transition. Microparticles also activated platelets and induced neutrophil migration *in vitro*. The inflammatory properties of microparticles involved activation of NF-κB and JNK, increased expression of E-selectin, P-selectin, ICAM-1 and VCAM-1. All these processes were blocked by coating microparticles with Diannexin.

**Conclusions:**

Following hepatic IRI, microparticles circulate and can be taken up by hepatocytes, where they activate signaling pathways that mediate inflammation and hepatocyte injury. Diannexin prevents microparticle formation and subsequent inflammation.

## Introduction

Hepatic ischemia-reperfusion injury (IRI) remains an important complication of liver surgery and transplantation [1], [2]. In the *early* phase (first 2 hr), hepatic damage is caused by oxidative stress, generated by Kupffer cells (KCs) with damage to sinusoidal endothelial cells (SECs) [1]–[6]. The *late* phase (6–24 hr) is mediated by hepatic recruitment of leukocytes and macrophages. These adhere to SECs that express adhesion molecules (E-selectin, P-selectin, ICAM-1, VCAM-1) and secrete chemokines [5]–[8] in response to hypoxia, oxidative stress, and tumor necrosis factor-α (TNF) [8]. Platelets are essential for hepatic IRI [9]; platelet clumps adhere to damaged SECs during the first 20 min of post-ischemic reperfusion [10]. The factors linking early SEC injury to inflammatory cell recruitment and platelet activation, with resultant microcirculatory impairment and hepatocyte injury have not been fully clarified.

Microparticles (MPs) are small (0.1–1.0 µm) fragments shed from blebbing of the outer leaf of plasma membranes of activated cells, such as platelets, macrophages, endothelial cells, lymphocytes, and hepatocytes, or from cells undergoing apoptosis [11], [12]. They are important in promoting inflammation and thrombotic responses following vascular dysfunction [11]–[16]. MPs harbour cell membrane proteins (used to identify their cellular origin) which also contain biologically active lipids. On their surface, MPs exhibit negatively charged phospholipids, chiefly phosphatidylserine (PS) which accounts for their pro-coagulant and pro-inflammatory properties [11]–[16]. Levels of circulating MPs increase in acute coronary ischemia, myocardial infarction and pre-eclampsia, likely contributing to the associated leukocyte and platelet adhesion and obstruction of microvascular blood flow [11]–[13].

Involvement of MPs in the pathogenesis of liver disease has been suggested in cirrhosis and hepatitis [17]–[19] but has not been reported so far in hepatic IRI. We have described extensive blebbing of SEC plasma membranes during the first 20 min of post-IR [10], leading us to conceive the hypothesis that MPs originating from perturbed plasma membranes of SECs contribute to the microvascular inflammatory and platelet activating responses in hepatic IRI. In the present work, we conducted experiments in a well-validated murine model of partial hepatic IRI [8], [10] to demonstrate time-dependent production of MPs in liver IRI, and to characterize their cellular derivation and lipid composition. We then determined the pathogenic effects of MPs, specifically their ability: (i) to cause hepatocyte injury directly, (ii) to activate platelets, (iii) to promote neutrophil recruitment, and (iv) to generate pro-inflammatory mediators, reactive oxygen species (ROS) and mitochondrial permeability transition (MPT)[7].

To pursue these aims we used a variety of complementary *in vitro* and *in vivo* approaches. We subjected primary hepatocytes to hypoxia-reoxygenation (HR) and showed that mitochondrial injury, activation of the c-jun *N*-terminal kinase (JNK) and TNF contribute to MP production from hepatocytes. Finally, we used the biosynthetic annexin V-homodimer, Diannexin (or ASP8597 as it is now referred to), which binds with high affinity to phosphatidylserine (PS) and prevents IRI [10], to show that this protein substantially reduces production of MPs *in vivo* following onset of post-ischemic reperfusion, and that residual Diannexin-coated MPs no longer exert pro-inflammatory or platelet-activating effects.

## Materials and Methods

### Murine model of partial hepatic IRI and administration of Diannexin

Groups (n = 10) of male C57BL6 mice age 8–12 weeks were anesthetized (ketamine 100 mg/kg, xylazine 20 mg/kg) and subjected to partial hepatic ischemia as reported [8], [10]. Temgesic 0.05 mg/kg is administered subcutaneously to animals on abdominal wound closure to provide post-operative analgesia during the recovery period. After 15 min–24 hr reperfusion, animals were again anesthetized were humanely killed by exsanguination. Blood and livers were collected [8], [10]. One group (sham) of control mice was subjected to anesthesia and sham laparotomy. Where indicated, Diannexin (1 mg/kg) (or saline vehicle) was administered via lateral tail vein (iv), 5 min prior to hepatic IRI. Protocols for N-acetylcysteine (NAC) pre-treatment in mice are described in detail in Materials and Methods S1.

### Ethics statement

The above animal protocols were approved by the Australian National University’s Animal Ethics Committee (protocol approval number #A2012/20) and conduct of experiments complied with the highest international criteria for humane care.

### Assessing severity of liver injury and cell viability

Liver injury was determined by serum alanine aminotransferase (ALT). In primary hepatocytes, lactate dehydrogenase (LDH) release was measured with the CytoTox-96-non-radioactive cytotoxicity assay (Promega, Madison, WI), and cell viability by 3-(4,5-dimethylthiazol-2-yl)-2,5-diphenyl-tetrazolium bromide (MTT) assay.

### Isolation of murine primary hepatocytes and liver leukocytes

Primary hepatocytes (>95% purity, >90% viability by trypan blue exclusion) were isolated from murine livers using collagenase perfusion (37°C; 8 mL/min), seeded (5 × 10^6^ cells/100 mm) in collagen 1-coated petri dishes and cultured in Williams-E (Gibco, St Louis, MO) supplemented with 10% heat-inactivated fetal bovine serum. To derive liver leukocytes, we used mechanical dissociation and centrifugation. Leukocytes were prepared in dextran (3%), then enriched for neutrophils by separation by Ficoll-Paque gradient, before semi-purified neutrophils were resuspended in RPMI. We simulated IRI *in vitro* using a hypoxia-reoxygenation (HR) chamber (Billrups-Rothenberg, CA) primed with 100% nitrogen for 15 min to establish total hypoxia. Reoxygenation was established by flushing with 21% O2/74% N2/5% CO2 (‘normoxia’). Control cells were cultured identically except for continued 21% O2/74% N2/5% CO2. Additions to primary hepatocytes in specific experiments (EGTA, SP600125) used to study processes such as MP formation and JNK activity are detailed in the figure legends.

### Preparation of MPs and flow cytometry analysis (FACS)

Blood was collected by cardiac puncture from anesthetized mice, spun for 15 min (1,500 g) and plasma re-centrifuged 2 min (13,000 g). MPs were sedimented from plasma supernatant by prolonged centrifugation (see Materials and Methods S1 for detailed MP isolation protocol). MPs were recovered from culture medium of primary hepatocytes using a similar protocol. Thereafter, MP pellets were resuspended in phosphate-buffered saline (PBS) before FACS analysis. We measured MP size using nanofluorescent polystyrene particle beads and FACS (Spherotech, IL). The gating strategy employed was based on size of MPs, 0.1 – 1 µm thereafter, analyses of MP subpopulations were carried out using cell-derived markers/tags (Table S1). FACS ARIA II (Becton-Dickinson, San Jose, CA) was utilized to sort MPs and FlowJo 2009 V.7.5 (Tree Star, Ashland, OR) software for analyses and generation of dot plots.

### PS detection by ELISA

MP production was determined semi-quantitatively by measuring phosphatidylserine (PS)-positive particles in the circulation (Zymuphen MP Activity, HYPHEN BioMed, France; see Materials and Methods S1). It is acknowledged that this method may also detect PS on apoptotic bodies. For this reason, we also employed a gated FACS strategy to isolate a purer fraction of MPs (or exosomes), as discussed later.

### Cellular origin of MPs and their bioactive lipids

To identify their cellular origin, MPs from mice subjected to IRI were freshly prepared in platelet-free plasma and incubated with the following cell-specific, FITC-labeled monoclonal antibodies: Annexin V, VE-cadherin (CD144), CD41, CD62P, CD1d tetramer, CD8, F4/80, Ly6G or isotype-matched control antibodies (Table S1). We used FACS to determine the relative proportions of each subset of cell-derived MPs. To assay MP bioactive lipid composition, pellets obtained after 60 min ischemia, 15 min and 2 hr reperfusion, were analyzed for F2-isoprostanes (lipid oxidation marker) and fatty acids by combination gas chromatography mass spectrometry (GCMS)/liquid chromatography tandem mass spectrometry (LC/MS/MS) [20], [21].

### Western blot analysis of ICAM-1, VCAM, E-selectin, P-selectin, VE-cadherin, ASGPR, IκB-α, COX-2, PKC-δ, JNK1/2, phosphorylated-JNK and measurement of serum TNF

20 µg of MP protein (Bradford assay) was resolved by 12% SDS-PAGE under reducing conditions, transferred to PVDF membranes and probed for the proteins detailed in Materials and Methods S1. Serum TNF was measured by ELISA (R & D Systems, Minneapolis, MN).

### Transwell migration assay

Neutrophil subpopulations of hepatic leukocytes were placed in 96 well chambers (ThinCert, Germany) fitted with porous PET membrane (8.0 µm pore diameter) inserts. The lower compartment was seeded with MPs, as specified in figure legends. In control wells (to correct for chemokinesis), equal concentrations of chemoattractants, IL-8 (10 ng/ml) or TNF (50 ng/ml) were added above and below the membranes. After incubation for 24 hr at 37°C, any non-migrated leukocytes were removed by washing with PBS twice and leukocytes that had migrated to the underside of the membrane, or which were present in the lower wells (stained with Giemsa-Wright) were counted. To ascertain effect of test compounds on transmigration potential, MPs were pre-treated with 1 mM *N*-acetylcysteine (NAC) or Diannexin (4 µg/ml) for 1 hr 37°C; thereafter, MPs were washed/resuspended in PBS then recovered by prolonged centrifugation twice (see Materials and Methods S1 for detailed MP protocol) prior to the transwell migration assay.

### Measurement of mitochondrial membrane potential (MPT) and oxidative stress

We used tetramethylrhodamine methyl ester (TMRM)(Sigma, St Louis, MO) fluorescence as a marker of mitochondrial membrane potential (ΔΨ_m_), 2,7-dichlororodihydrofluorescein diacetate (DCFH2-DA, Sigma, St Louis, MO) fluorescence for oxidative stress (Materials and Methods S1). The assay was performed using William’s E media in view of primary hepatocyte cultures studied; the solvent for DCFH2-DA was PBS at a final concentration of 10 µM.

### Statistical analyses

Analysis of variance (ANOVA) with Tukey post-hoc analysis was used for the comparison of data from different experimental groups. Results presented as mean ± SD, significant *P*<0.05.

## Results

### PS-containing MPs shed during post-ischemic reperfusion bear markers of endothelial cells, platelets, inflammatory cells and hepatocytes

**Figure 1:**
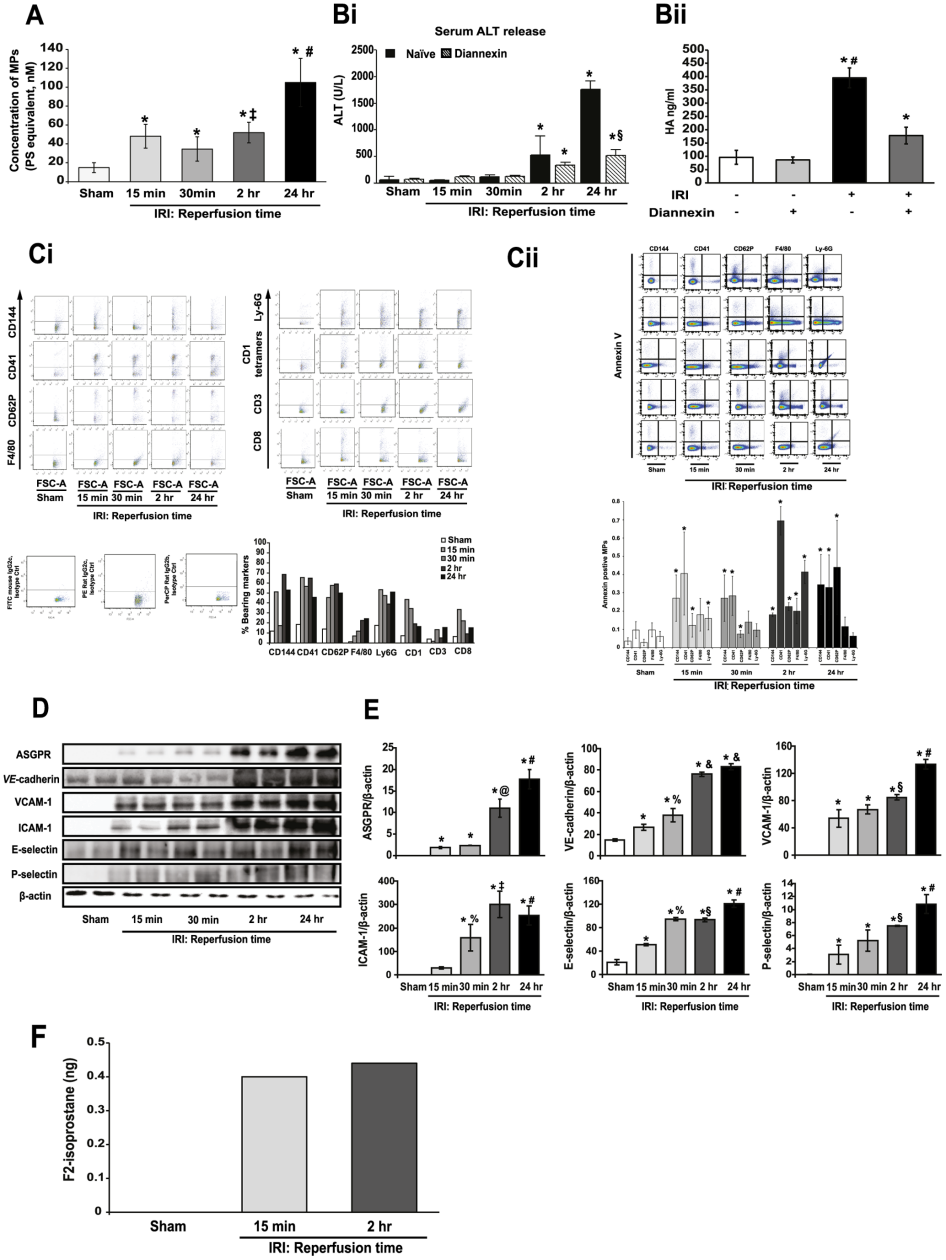
MP production is increased in hepatic IRI. **A**. MP release (concentration expressed as phosphatidylserine, PS equivalent in nM) and **Bi,ii** serum ALT and hyaluronic acid (HA) after 60 min ischemia and indicated reperfusion times in naïve and Diannexin-treated mice (n = 10 per cohort). * p<0.05 all experimental groups vs. sham. ‡ p<0.05 IRI 2 hr vs. 30 min reperfusion. # p<0.05 IRI 24 hr vs. 15 min, 30 min and 2 hr reperfusion. § p<0.05 Diannexin vs. no Diannexin at 24 hr reperfusion. **C**. FACS plots demonstrating MPs released early in reperfusion are composed predominantly of endothelial-derived/CD144 remnants while in late reperfusion, MPs are largely from leukocytes and hepatocytes. (**Ci**) FACS data depicted as % bearing cell-specific markers (Table S1). Note, because individual MPs are cell fragments and may potentially fuse, MPs can bear more than 1 cell-marker and therefore, the sum of all expressed markers may feasibly exceed 100% (Materials and Methods S1). (**Cii**) Annexin V was utilized to analyze for double positive events together with cell-specific markers CD144, CD41, CD62P F4/80 and Ly6G. * p<0.05 IRI groups vs. sham. **D**. MPs bear ASPGR (hepatocytes) and VE-cadherin (SECs), VCAM-1, ICAM-1, E-selectin, P-selectin. MPs obtained from mice subjected to liver post-IR at indicated times. Blots representative of three similar experiments. **E**. Image analyses/quantification show upregulation of adhesion molecules especially at 2, 24 hr reperfusion. * p<0.05 IRI vs. sham. & p<0.05 IRI 2 and 24 hr vs. IRI 15 and 30 min reperfusion. %p<0.05 IRI 30 min vs. IRI 15 min reperfusion. # p<0.05 IRI 24 hr vs. 15,30 min, 2 hr reperfusion. § p<0.05 IRI 2 hr vs. 15 min reperfusion. ‡ p<0.05 IRI 2 hr vs. IRI 30 min reperfusion. **F**. MPs at 15 min and 2 hr reperfusion contain increased levels of F2-isoprostanes compared to those derived from sham-operated mice by GCMS and LC/MS/MS (normalised to total AA detected).

By an assay which quantifies surface PS, we demonstrated a significant increase in MP production during post-ischemic reperfusion from 15 min, before serum ALT increased (Fig.1A,Table 1). We acknowledge that this assay would also determine PS-positive apoptotic fragments (see later comment). MP production/detection persisted through the early and late phases of hepatic IRI (30 min, 2 hr) to reach peak plasma levels at 24 hr reperfusion (Table 1). The time discrepancy between early increases MP and eventual rise in ALT levels (Fig.1Bi) indicates that the production of MPs precedes and does not simply reflect hepatic injury in IRI. We also measured serum hyaluronic acid (HA, Materials and Methods S1) whose increase partially reflects the degree of SEC dysfunction or release from the surface glycocalyx of SECs during post-ischemic reperfusion. Following 60 min ischemia and 15 min reperfusion, serum HA increased strikingly compared to sham-operated mice (Fig.1Bii) reflecting very early release from SECs during reperfusion.

**Table 1:** Microparticles released at indicated reperfusion time after 60 min hepatic ischemia in naïve and Diannexin-treated mice (n = 10 per experimental group).

Experimental group, and reperfusion time	Microparticles released (PS equivalent, nM)	
	Naïve	Diannexin
Sham	14.7±5.52	12.5±1.57
15 min	48.1±12.6 *	29.9±3.25 * ^@^
30 min	34.7±12.8 *	20.1±5.77 ^&^
2 hr	51.9±10.9 *	35.2±3.42 * ^#^
24 hr	104±25.6 *	44.8±9.43 * §

*p<0.05 all experimental groups vs. sham.

@ p<0.05 Diannexin vs. 15 min reperfusion.

& p<0.05 Diannexin vs. 30 min reperfusion.

# p<0.05 Diannexin vs. 2 hr reperfusion.

§ p<0.05 Diannexin vs. 24 hr reperfusion.

In order to establish the nature of MPs that circulate during hepatic IRI, we first used FACS gating to determine their size. We then partially characterized their cells of origin by incubation with fluorescent-labeled mAbs directed at molecules specific or relatively specific (in the case of CD62P) to different cell types [22](Table S1). MPs obtained post-IRI varied from 0.45 - 1 µm (Fig.1C). MPs appeared to be released in a time-dependent manner during post-ischemic reperfusion (Fig.1A), and like serum ALT increase, reached peak levels at 24 hrs reperfusion (Fig.1A,1Bi). MPs produced *early* (15, 30 min) in reperfusion were enriched for endothelial cell (CD144, CD62P for activated SECs), platelet (CD41, CD62P also for activated platelets) and neutrophils (Ly6G) markers, indicating predominant origins from SECs, platelets and neutrophils. In addition, NK-T cell (CD1d tetramer) and CD8 T-cell markers were also expressed (Fig.1Ci). By 2 hr reperfusion, markers of Kupffer cells/macrophages (F4/80) were more conspicuous on MPs (Fig.1Ci,ii), while NK-T cells and CD8 markers diminished (Fig.1Ci). To confirm co-location of cell-specific markers with 0.45 – 1 µm MPs (excluding most apoptotic bodies – see earlier), we established that subsets of MPs released post-IR double stained positive for Annexin V, as well as CD144, CD41, CD62P, F4/80 and Ly6G (Fig.1Cii).

As an additional approach to MP cell-source characterization, we performed immunoblot analysis of MP proteins. At 15 min reperfusion, VE-cadherin, E-selectin (endothelial cell proteins) and vascular adhesion molecule (VCAM-1) were detected (Fig.1D,E). By 30 min, P-selectin (platelets) and ICAM-1 were also detectable. At 2 hr, all the above proteins were substantially enhanced in MPs, with a dramatic increase in hepatocyte-specific asialoglycoprotein-receptor (ASGPR) as well as VE-cadherin (Fig.1D,E).

### MPs shed during post-ischemic reperfusion contain bioactive lipids

As expected from their plasma membrane origin, MPs contain lysophosphatidylcholine (lysoPC), arachidonic acid (AA) metabolites and oxysterols [23]-[25]. Because of their potential relevance to inflammation and coagulation, we assayed the following bioactive lipid species within MPs obtained during the early phase (15 min, 2 hr) of post-ischemic reperfusion: (i) AA metabolites – prostaglandins, leukotrienes, hydroxyeicosatetraenoic acid, epoxyeicosatrienoic acids, F2-isoprostanes, (ii) specific phospholipids namely lysoPC, platelet activating factor (PAF), lysoPAF, PS, PE, and (iii) cholesterol oxidation products (oxysterols). At both reperfusion times, samples contained a range of fatty acids (palmitic, stearic, oleic, linoleic). Whereas MPs at 15 min (ultra-early) reperfusion contained only a trace of AA, those at 2 hr were enriched with AA (3.6 µg total or 6 µg/mg protein). Samples also contained increased F2-isoprostanes derived from free radical oxidation of AA: at 15 min, 0.40 ng; 2 hr, 0.44 ng (Fig.1F) compared to those derived from sham-operated mice. Thus, MPs released during the first 2 hr of post-ischemic reperfusion contain several lipid molecules known to trigger inflammation and injury.

### MPs generated during hepatic IRI promote neutrophil migration and activate platelets

**Figure 2:**
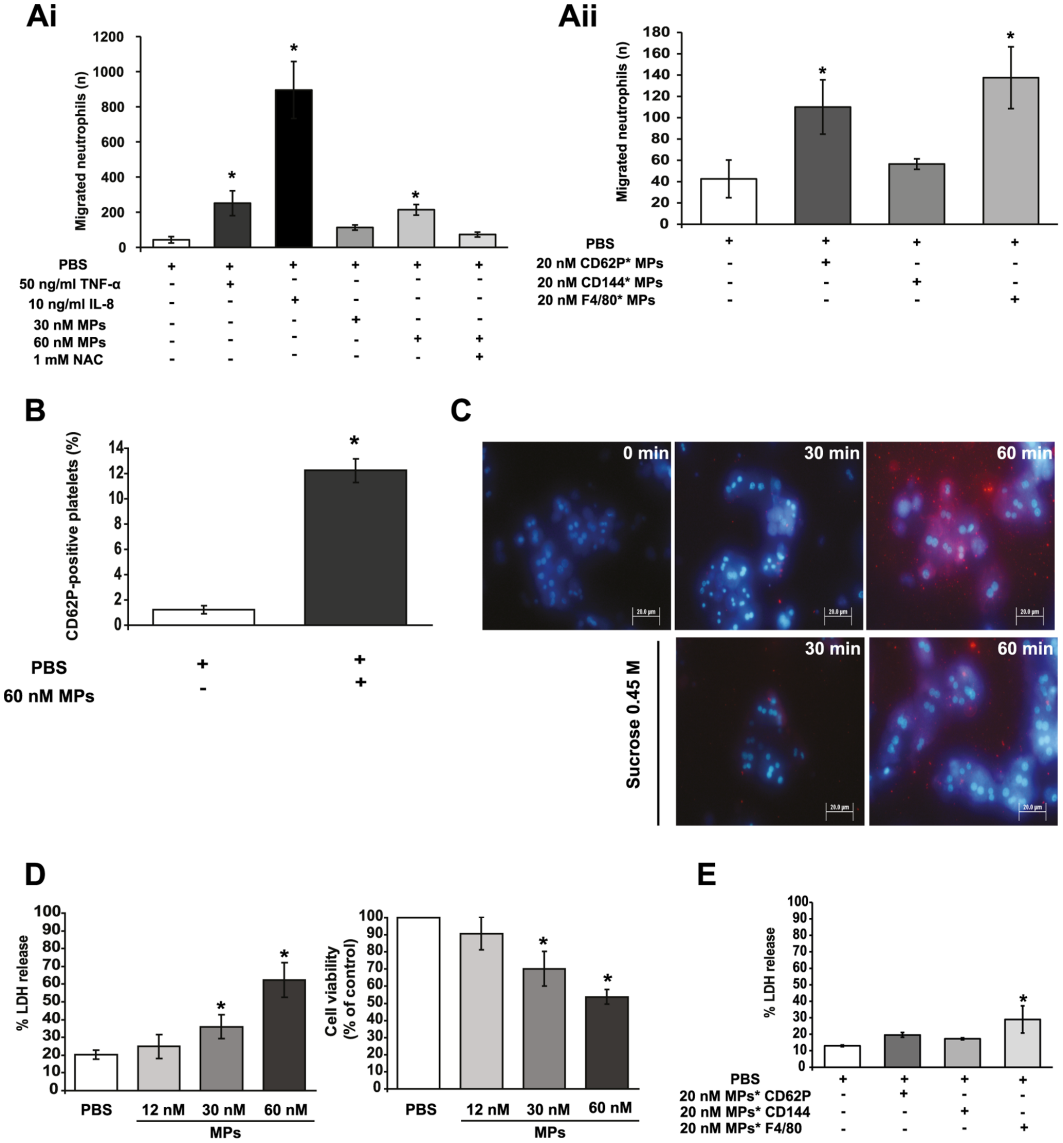
MPs are directly pathogenic to primary hepatocytes, incite neutrophil recruitment and activate platelets. **Ai**. Hepatic neutrophils were derived by liver perfusion and chemotaxis measured in ThinCert chambers. Lower compartments were seeded with MPs from mice subjected to 2 hr post-ischemia-reperfusion (IR), IL-8 or TNF-α as chemoattractants (IL-8,TNF:positive controls) and incubated (1 hr, 37°C). Co-treatment of MPs with 1 mM NAC reduced neutrophil transmigration. To ascertain effect of *N*-acetylcysteine (NAC) on transmigration potential, MPs were pre-treated with 1 mM NAC for 1 hr 37°C; thereafter, MPs were resuspended in PBS then recovered by prolonged centrifugation (see Materials and Methods S1 for detailed MP isolation protocol) prior to the transwell migration assay. **Aii.** Addition of MPs generated by IRI from specific cell types (significantly, activated platelets and endothelial cells, CD62P and macrophages, F4/80; not resting SECs, CD144) to these chambers enhanced transmigration of leukocytes. * p<0.05 experimental groups vs. control. **B**. Platelets from sham-operated mice treated with 60 nM MPs (from mice subjected to 2 hr post-IR) for 30 min, labelled with CD62P (activated platelet and endothelial cell marker) and subjected to FACS. * p<0.05 experimental groups vs. control. **C**. MPs from mice subjected to 2 hr post-IR co-incubated with primary hepatocytes. By 30 min, MPs adhered to hepatocytes and were endocytosed at 1 hr (first row/top panel of figures: 0 min, 30 min, 60 min); engulfment was inhibited by 0.45 M sucrose (second row/lower panel of figures: 30 min, 60 min). **D**. Primary hepatocytes treated with increasing concentrations of MPs derived from mice subjected to 2 hr post-IR. LDH leakage and cell viability (MTT assay) in MP-treated cells compared with PBS-controls. (**E**) Primary hepatocytes treated with increasing concentrations of MPs derived from specific cell types (CD62P: activated platelets and endothelial cells, CD144: SECs, F4/80: macrophages) obtained from mice subjected to 2 hr post-IR. Experiments performed in triplicate in MP-preparations from 4 mice (n = 12). *p<0.05 experimental groups vs. control.

In order to test our hypothesis that MPs originating from SECs, inflammatory cells and platelets incite the early inflammatory/injury response in IRI [10], [17], we tested whether circulating MPs isolated in these experiments could to promote neutrophil recruitment. Addition of MPs generated by IRI (Fig.2Ai), as well as those from specific cell types (Fig.2Aii, particularly CD62P: activated platelets, F4/80: macrophages) to these chambers enhanced transmigration of leukocytes obtained from normal mouse liver in a dose-dependent manner. In contrast, MPs pre-treated with *N*-acetylcysteine (NAC), a ROS and reactive nitrogen species scavenger, inhibited leukocyte transmigration in response to MPs (Fig.2Ai). Because the overall neutrophil responses elicited by cell-specific subtypes of MPs were similar to those from the ‘mixed’ pool of MPs derived at 2 hr reperfusion, we used the latter for subsequent studies. Such MPs activated platelets derived from sham-operated mice as shown by enrichment of CD62P (activated platelets) in relation to CD41 (all platelets)[26] compared to PBS-treated controls (Fig.2B). Collectively, these findings are consistent with our hypothesis that MPs liberated during liver IR from SECs and other cell types induce neutrophil migration and activate platelets.

### MPs generated during hepatic IRI induce injury in primary hepatocytes by a process that involves oxidative stress and mitochondrial membrane permeability transition

Because of their bioactive lipid content and potentially reactive oxygen intermediates and other pro-oxidants, MPs could also be directly cytopathic to hepatocytes. If so, this could, at least partially, contribute to the presence of hepatocyte-specific markers (ASGPR-positive) on MPs that circulate 2 and 24 hr post-IR. To test this, we incubated primary murine hepatocytes (cultured in Williams E media) with MPs isolated from mice subjected to ischemia/2 hr reperfusion. Within 30 min, these MPs adhered to hepatocytes, while endocytosis clearly evident by 1 hr (Fig.2C). This uptake process was inhibited by hypertonic sucrose (0.45 M) which blocks endocytosis (Fig.2C)[27]. Following engulfment of MPs (and not in control hepatocytes to which vehicle alone was added), hepatocyte injury was evident by LDH leakage (Fig.2D). Such MP-induced hepatocyte injury was particularly striking for F4/80 positive-MPs, which is consistent with the proposed role of KCs in hepatic IRI (Fig.2E).

**Figure 3:**
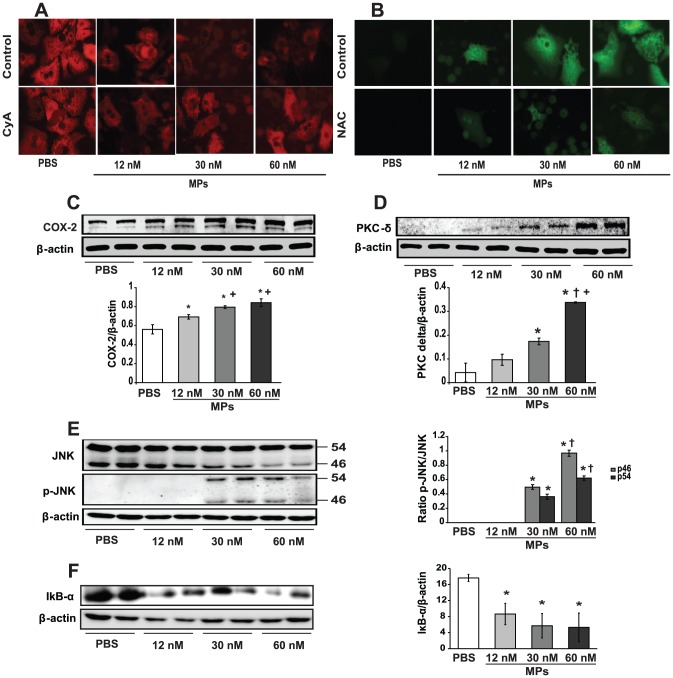
MPs invoke mitochondrial permeability transition, oxidative stress, adhesion molecule expression and activate NF-κB in hepatocytes. **A**. Hepatocytes loaded with 50 nM TMRM (red fluorescence), 1 µM DCFH2-DA (green fluorescence) and placed in chambers at 37°C for 15 min. MPT occurred with increasing MP concentrations, blocked by 1 µM CyA. **B**. 1 mM of NAC inhibited oxidative stress generated by MPs. **C, D**. Primary hepatocytes were exposed to 60 nM of MPs derived from liver 2 hr post-IR and pro-inflammatory molecules COX-2 and PKC-δ determined (WB) as well as (**E**) JNK-1/2 (46 kDA, 54 kDA) activation (phospho-JNK). * p<0.05 experimental groups vs. control. + p<0.05 30 and 60 nM vs. 12 nM MPs. † p<0.05 MPs 60 nM vs. 30 nM MPs. **F**. MPs activate NF-κB in primary hepatocytes, by IκB-α degradation (immunoblots). Blots represent three experiments conducted in triplicate (n = 9). * p<0.05 experimental vs. control.

To elucidate the mechanism by which IRI-related MPs injure hepatocytes, we used confocal microscopy of TMRM fluorescence [28] to show how addition of MPs caused MPT, a phenomenon blocked by cyclosporine A (CyA)(Fig.3A). Further, we observed evidence of oxidative stress in MP-treated primary hepatocytes which could be abrogated by the antioxidant, N-acetylcysteine (NAC) in DCFH2-DA studies (Fig.3B). Thus, MPs induce oxidative stress in hepatocytes and provoke MPT whilst primary hepatocytes can be ‘rescued’ from MP-related injury by co-treatment with an antioxidant or MPT inhibitor.

The effect of NAC on MPs release was further clarified by administering this potent antioxidant to mice (150 mg/kg ip, Materials and Methods S1) 5 min prior to 60 min ischemia and 24 hr reperfusion. NAC significantly inhibited MP release and was profoundly hepatoprotective against IRI compared to naïve mice (Fig.S1A,B). Moreover, NAC greatly diminished the oxidative potential of MPs in primary hepatocytes and *in vivo*, as shown by a marked reduction in glutathione oxidation (oxidized glutathione/reduced glutathione, GSSG/GSH, Fig.S1C,D).

### MPs initiate post-ischemic inflammatory responses by activating NF-κB in hepatocytes

To further define their pro-inflammatory potential, MPs from mice subjected to IRI (as well as those derived from primary hepatocytes subjected to hypoxia-reoxygenation, HR, described later), were probed for adhesion molecules. As mentioned earlier, MPs isolated at 15 min reperfusion bear appreciable VCAM-1, E-selectin and P-selectin, while ICAM-1 is expressed on MPs shed at 30 min reperfusion, and VE-cadherin is detected at 2 and 24 hr (Fig.1D,E). Thus, the profile of pro-inflammatory adhesion molecules borne on MPs differs with reperfusion time. Further, it recapitulates the suite of different adhesion molecules known to be expressed in livers subject to IRI [1]–[6].

Addition of MPs to primary hepatocytes likewise induced ICAM-1 and VCAM-1, and up-regulated pro-inflammatory COX-2 and PKC-δ protein expression (Fig.3C,D). MPs also activated c-jun *N*-terminal kinase (JNK)(Fig 3E), a known mediator of hepatocyte injury in IRI [1]–[6]. To establish the effects of MPs on NF-κB activation, we determined degradation of cytosolic IκB-α, and show that MPs caused a dose-dependent decrease in IκB-α (Fig.3F).

### Hypoxia-reoxygenation leads to MP formation from primary hepatocytes by an oxidative stress and calcium-dependent process

**Figure 4:**
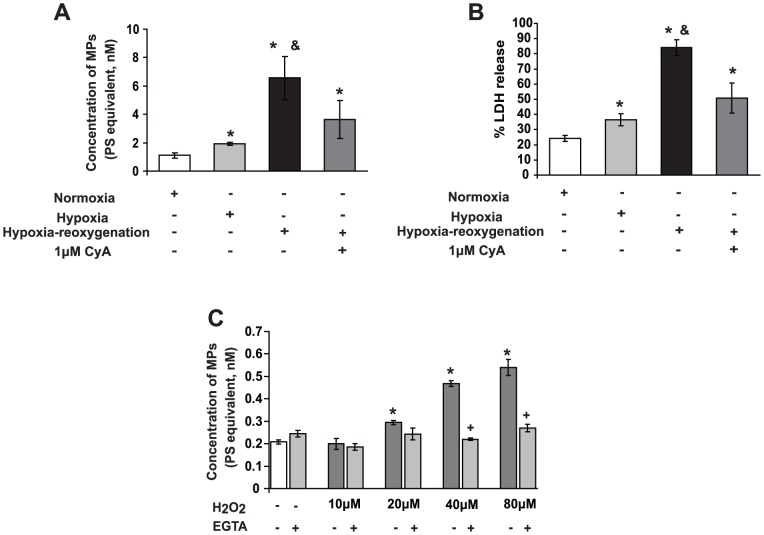
Hypoxia-reoxygenation and oxidative stress trigger MP release. **A**. Primary hepatocytes were exposed to 4 hr hypoxia followed by 24 hr reoxygenation (Methods). Pretreatment with 1 µM CyA (MPT-inhibitor) for 2 hr prior to HR attenuated MP release and (**B**) LDH leakage from hepatocytes subjected to HR. Controls: cells incubated in normoxic conditions. Experiments performed in triplicate. * p<0.05 experimental vs. control. & p<0.05 hypoxia-reoxygenation vs. CyA. **C**. H_2_O_2_ causes MP primary hepatocytes to form MPs, which is attenuated by calcium chelation (EGTA). Primary murine hepatocytes were incubated with H_2_O_2_ for 24 hr. At 10 µM or higher, H_2_O_2_ causes generation of MPs, while MP release is abolished by co-administration of EGTA. Assays performed in triplicate. * p<0.05 experimental vs. control. +p<0.05 EGTA vs. H_2_O_2_.

In order to simulate the *in vitro* conditions for MP formation relevant to hepatic IRI *in vivo*, we exposed primary hepatocytes to 4 hr hypoxia followed by 24 hr reoxygenation. This liberated MPs, whereas hepatocytes subjected to normoxia alone did not (Fig.4A). CyA significantly diminished MP release after HR and markedly reduced cell injury (Fig.4B). To establish more directly that oxidative stress is responsible for MP generation, we incubated primary murine hepatocytes from mice with escalating doses of H_2_O_2_. At 20 µM or higher, H_2_O_2_ caused generation of MPs from primary hepatocytes (Fig.4C), a process abrogated by addition of 1 mM EGTA, a potent Ca^2+^ chelator [28], thereby abrogating activity of Ca^2+^–dependent proteases such as calpain. These findings suggest that MP release from hepatocytes is triggered by oxidative stress and is Ca^2+^-dependent.

### TNF augments JNK-dependent MP formation by primary hepatocytes, as well as in mice after IRI

**Figure 5:**
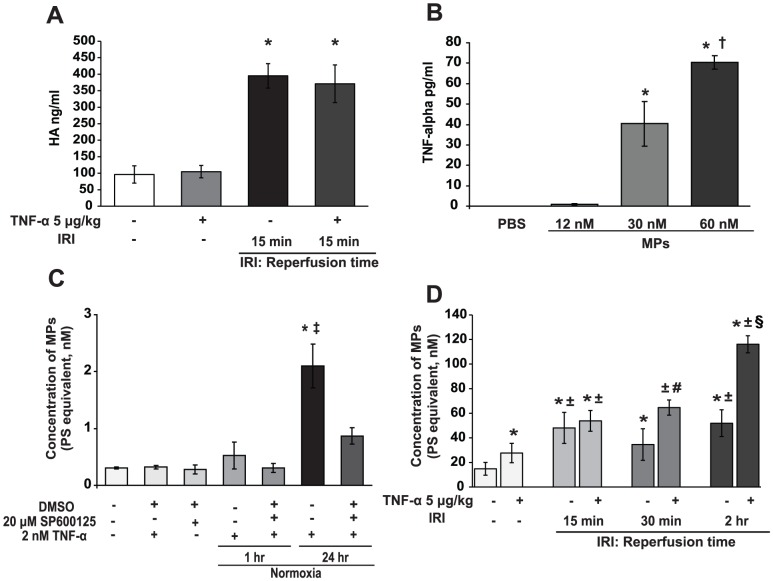
MPs stimulate TNF release and are themselves potent agonists of MP vesiculation *in vivo*. **A**. Serum hyaluronic acid (HA) release increases in mice subjected to 60 min ischemia and 15 min reperfusion; this is exacerbated by administration of TNF *in vivo* (mice injected with TNF 5 µg/kg iv vs. saline controls), 5 min prior to 60 min ischemia. * p<0.05 experimental vs. control. **B.** TNF is produced by primary hepatocytes following addition of MPs derived from mice subjected to 2 hr post-ischemic reperfusion. * p<0.05 experimental vs. control. † p<0.05 60 nM vs. 30 nM MPs. **C**. Primary hepatocytes were incubated with 2 nM TNF for 1 hr and 24 hr in normoxic conditions. SP600125 (20 µM in 0.1% DMSO) significantly attenuated TNF-stimulated MP release. * p<0.05 experimental vs. control. ‡ p<0.05 TNF vs. TNF+SP600125. **D**. WT CL57BL6 mice were injected i.v. with TNF 5 µg/kg (saline for controls), 5 min prior to 60 min ischemia. MP production was measured in plasma at 15 min, 30 min, 2 hr reperfusion. Liver IRI triggered MP production; TNF injection further augmented MP release reaching a maximum at 2 hr. * p<0.05 experimental vs. control. ± p<0.05 IRI groups vs. sham TNF. # p<0.05 30 min+TNF vs. IRI 30 min reperfusion. § p<0.05 2 hr+TNF vs. 2 hr reperfusion.

TNF levels increase strikingly during liver IR, and an injection of high dose TNF prior to IR exacerbates liver injury [2], [8] as well as SEC dysfunction by serum hyaluronic acid release (Fig.5A). TNF is a potent agonist of endothelial MP vesiculation and contributes to MP production *in vitro* [29]. It is therefore of interest to establish whether MPs stimulate TNF release *in* primary hepatocytes. As shown in Fig.5B, addition of 30 and 60 nM of MPs for 1 hr under normoxic conditions provoked at least 4-fold stimulation of TNF production from hepatocytes. Conversely, incubating primary hepatocytes with 2 nM TNF triggered perceptible MP production at 1 hr, increasing significantly by 24 hr, by a process abrogated by addition of SP600125 at concentrations for which it is a specific JNK inhibitor (Fig.5C). Finally, to establish whether TNF has similar effects on MP release *in vivo*, we injected murine TNF intravenously (5 µg/kg) 5 min prior to onset of 60 min ischemia, then measured MP production. The results show that TNF augments MP production *in vivo* during hepatic IR (Fig.5D), reaching a maximal response at 2 hr reperfusion.

### The annexin V-homodimer, ASP8597 (Diannexin) inhibits MP production after hepatic IRI and reduces platelet activation

**Figure 6:**
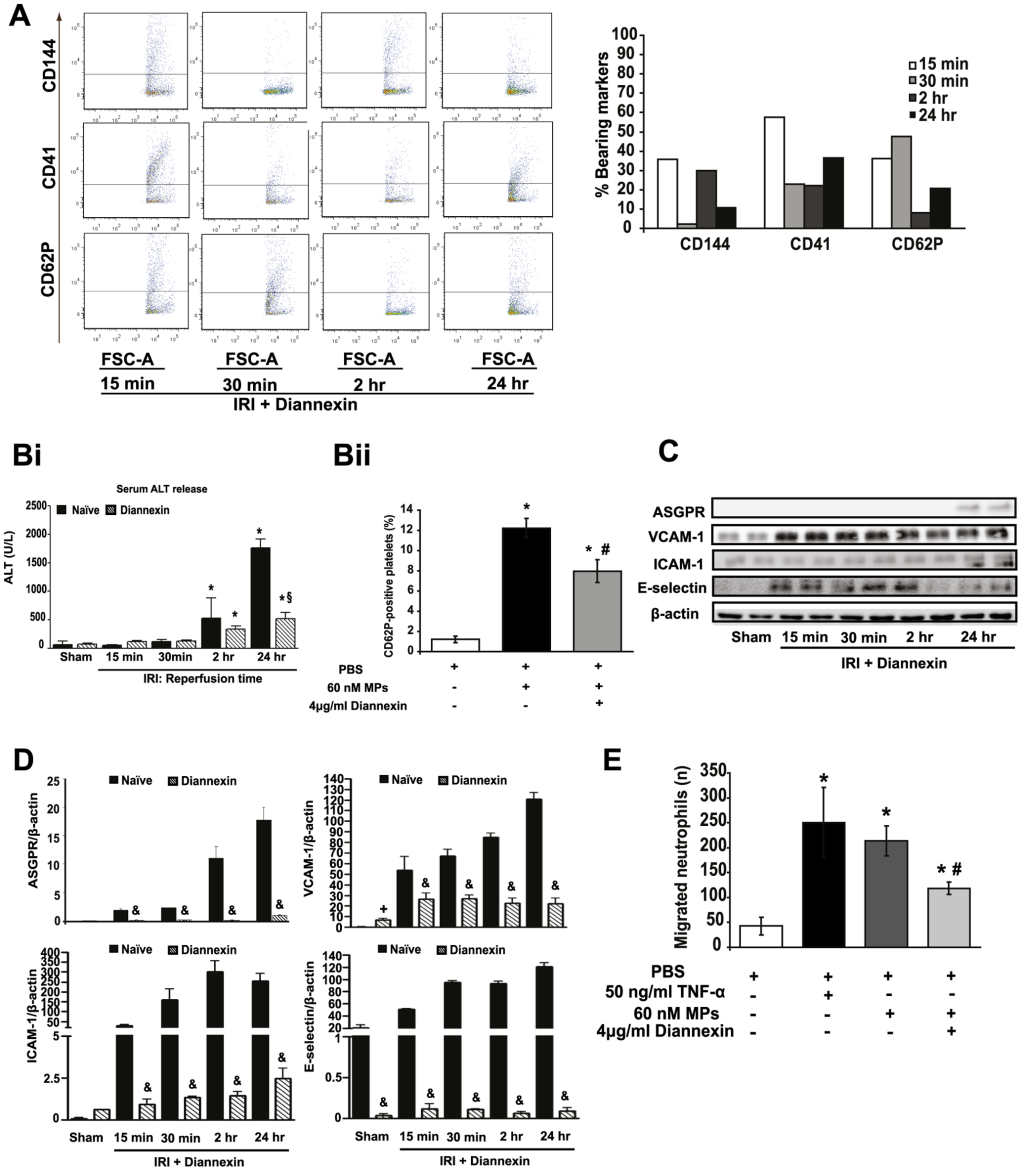
Diannexin attenuates release of MPs after hepatic IRI and decreases their pro-inflammatory and deleterious potential. MPs were obtained from mice pre-treated with 1 mg/kg iv Diannexin 5 min prior to 60 min liver IR and results compared with vehicle-treated/naïve mice subject to IR. **A**. FACS revealed significantly reduced resting endothelial cell (CD144), resting platelet (CD41) and activated platelet and endothelial cell (CD62P) subsets compared to naïve (see Fig.1C). **Bi**. Serum ALT after 60 min ischemia and indicated reperfusion times in naïve and Diannexin-treated mice (n = 10 per cohort). * p<0.05 all experimental groups vs. sham. § p<0.05 Diannexin vs. 24 hr reperfusion. **Bii**. Platelets were isolated from sham-operated mice and exposed to 60 nM MPs (derived from mice subjected to 60 min ischemia, 2 hr reperfusion) in the absence or presence of 4 µg/ml Diannexin. FACS then performed using CD62P to quantify activated platelet subset as a proportion of total platelets. * p<0.05 experimental vs. control. # p<0.05 Diannexin vs. 60 nM MPs. **C, D**. Hepatocyte-specific ASPGR, E-selectin and pro-inflammatory VCAM, ICAM-1 protein expression diminished by Diannexin compared to naïve mice subjected to 60 min ischemia and indicated reperfusion times. + p<0.05 Diannexin vs. sham. & p<0.05 Diannexin vs. naïve. **E**. Pre-treatment of MPs with Diannexin 4 µg/ml inhibited neutrophil transmigration in ThinCert chambers. To ascertain effect of test compounds on transmigration potential, MPs were pre-treated with Diannexin (4 µg/ml) for 1 hr 37°C; thereafter, MPs were resuspended in PBS then recovered by prolonged centrifugation twice (see Materials and Methods S1 for detailed MP isolation protocol) prior to the transwell migration assay. * p<0.05 experimental vs. control. # p<0.05 Diannexin vs. 60 nM MPs.

Because Annexin V efficiently binds to MPs (most likely to everted PS residues)[22], we investigated whether such a strategy would also block MP production (Fig.6A) and prevent hepatic IRI. Diannexin is a biosynthesized human recombinant homodimer of annexin V that exerts considerable hepatoprotection against IRI in our murine model (Fig.6Bi); its efficacy associated with reduced swelling and detachment of SECs from their basement membrane, decreased hepatic expression of pro-inflammatory ICAM-1, VCAM and MIP-2, and abrogated leukocyte and platelet adherence to SECs [9], [10]. To establish whether this protective action involves attenuation of MP release, we injected mice with Diannexin (1 mg/kg), 5 min prior to hepatic ischemia and measured MP production during reperfusion. At all timepoints, Diannexin significantly diminished MP release, liver and SEC injury (by serum ALT and HA respectively) compared to naïve mice (Fig.6Bi,1Bii, Table 1). By FACS, Diannexin not only substantially reduced MP production, it also altered the profile of cell markers detected on the residual trivial quantities of MPs derived (Fig.6A *vs* Fig.1C). Thus, these residual MPs displayed reduced markers for endothelial cells (CD144), platelets (CD41) and activated platelets (CD62P)(Fig.6A) confirming our proposal that the major effect of Diannexin is on SECs and platelets, and occurs early during post-ischemic reperfusion [10]. Further, the protective efficacy and suppression of MP production conferred by Diannexin is sustained into late reperfusion (Fig.6Bi).

Incubation of platelets derived from sham-operated mice with MPs isolated from mice after 60 min ischemia/2 hr reperfusion resulted in a significant induction of activated (CD62P+ve) platelets (Fig.6Bii). However, if such MPs were “Diannexin-treated” (4 µg/ml) then exposed to ‘normal’ platelets *in vitro*, this strategy yielded significantly reduced populations of CD62P+ve platelets compared to those exposed to “Diannexin-naïve” MPs (Fig.6Bii)

### Diannexin blocks the pro-inflammatory and pro-oxidant properties of MPs released during hepatic IRI

While Diannexin reduced total MP production after IRI, the attendant abrogation of liver injury by ALT (Fig.1Bi) and histology was even more complete [10]. This would be explained if MP production was only part of the hepatoprotective effect of Diannexin, or if residual MPs failed to exert their pro-inflammatory and platelet-activating properties. To test the latter proposal, MPs from Diannexin-treated animals subjected to IRI were analyzed for the expression of hepatocyte-specific marker ASGPR, and inflammatory adhesion molecules, VCAM-1, ICAM-1 and E-selectin by immunoblotting. MPs from Diannexin-treated mice yielded significantly reduced markers for cells of hepatocyte origin (Fig.6C,D). While VCAM-1, ICAM-1 and E-selectin were strikingly upregulated in MPs released in naïve mice during late reperfusion (24 hr)(Fig.1D,E), their expression was significantly mitigated after Diannexin treatment (Fig.6C,D). In *in vitro* experiments, incubation of MPs isolated from mice after 60 min ischemia/2 hr reperfusion with Diannexin *in vitro*, markedly inhibited leukocyte transmigration potential in chemotaxis studies (Fig.6E).

The oxidation status of MPs derived from Diannexin-treated mice was determined by oxidized glutathione/reduced glutathione (GSSG/GSH) measurements. As depicted in Fig.S1C, glutathione oxidation was significantly diminished by Diannexin-pretreatment compared to levels in MPs derived from naïve mice. Taken together, this suite of interventions confirm that Diannexin effectively abrogates cell-specific injury particularly to SECs, hepatocytes, reduces platelet-activation, the pro-inflammatory and pro-oxidant properties of MPs liberated during liver IR and that these findings strongly correlate with hepatoprotection by serum ALT (Fig.6Bi).

## Discussion

MPs are cell membrane fragments shed upon injury or apoptosis whose circulating levels have been reported as biomarkers for such vasculopathies as acute myocardial infarction, ischemic stroke, peripheral vascular disease and eclampsia [11]–[13]. In the present study, we first established that MPs are liberated during hepatic IRI, arising in the earliest stages from activated or injured SECs, and in the later phases, involving platelets, various inflammatory cells and eventually hepatocytes. We then demonstrated that MPs actively contribute to the pathogenesis of IRI via their well-known pro-inflammatory effects and their role as platelet-activating agents. We also provided evidence for an entirely novel pathway of direct hepatocyte injury in IRI. Finally, our novel findings clarify the mechanism for the powerful therapeutic efficacy of the annexin V dimer, Diannexin, which would be expected to “seek and hide” exposed PS residues on circulating MPs [10].

MPs are released within minutes of IR or hypoxia-reoxygenation. This is followed by further cascades of shedding from a variety of cell types over several hours. Although the present data are confined to small MPs (0.45 – 1 µm) that comprise exosomes rather than apoptotic bodies, it is possible that larger MPs (aptosomes) also play a role in IRI, as might lipid rafts and smaller MPs. This requires further study using newer technology for MP purification. However one of the major new findings of the present studies is that circulating MPs are engulfed by hepatocytes, and this causes ROS generation with induction of MPT as a pathway to cell injury and necrotic cell death. In keeping with reports in other cell types [12], [13], our results also provide direct evidence that hepatocyte-derived MPs cause dose-dependent TNF release from other hepatocytes, a process that is ROS-dependent and can be abrogated by NAC *in vivo* and *in vitro*. This is potentially important in propagation of liver IRI as TNF further amplifies MP shedding *in vivo* during hepatic IRI, also by a redox and calcium-dependent process. Interruption of TNF release with NAC, an agent already in use in clinical practice for acetaminophen overdose, so as to mitigate propagation of liver IRI is worthy of further study in humans after hepatic surgery or liver transplantation.

The phospholipid composition of each leaflet of the plasma membrane bilayer differs; PS and PE aggregate in the inner leaflet, PC and sphingomyelin are present in the outer layer [17]. Loss cellular energy, or after cytoskeletal disruption (as in apoptosis) leads to loss of membrane integrity with eversion of the negatively charged PS and PE. Hence, MPs liberated after cellular injury usually bear PS on their extruded surfaces. Other lipid molecules with biological activity are contained within MPs including lysoPC, AA metabolites (including F2-isoprostanes) and oxysterols. Both the nature of cellular insult and the cell type of origin influence MP constituents [11], [22]. In our studies, MPs generated after 15 min reperfusion contained only a trace of AA, but the amount escalated by 2 hr reperfusion, and at this time highly pro-inflammatory F2-isoprostanes could also be detected. The nature of our studies in the whole mouse does not allow clarification of why MP lipid composition changes post-ischemic time, other than to note that AA and F2-isoprostanes which were detected at 2 hr but only in vanishingly small quantities at 15 min, were more likely to arise from platelets and macrophages rather than SECs, other inflammatory cells and hepatocytes.

In order to understand how MPs could exert pro-inflammatory effects, as demonstrated by the neutrophil migration studies, we probed MP pellets for adhesion molecules. As early as 15 min reperfusion, circulating MPs express VCAM-1, E-selectin and P-selectin (Fig.1D), while ICAM-1 and VE-cadherin were detected later. Further, our *in vitro* approaches demonstrated unambiguously that the MPs which circulate during post-ischemic reperfusion with the most potent pro-inflammatory properties are KC/macrophage- and platelet-derived. The present results clearly indicate that MPs engulfment by hepatocytes leads to degradation of IκB-α, the cytosolic inhibitor of NF-κB. Activation of NF-κB in livers subjected to IR upregulates numerous pro-inflammatory genes such as COX-2 and PKC-δ that participate in liver inflammatory recruitment and cell death [2], [3], [5], [6]. These effects were observed in the context of MP stimulation, together with elaboration of TNF and activation of JNK, both of which are known pro-inflammatory pathways in hepatic IRI injury [8], [30]. Thus, in addition to direct effects of redox stress, TNF and JNK activation, MP engulfment is another node in the pathways to pro-inflammatory signaling during IRI. Importantly, as already mentioned, this is another site at which NAC could exert therapeutic effects.

The formation of membrane-derived MPs is dependent on increased intracellular ionic calcium which activates calpain. We and others have shown that hepatic calpain activity escalates with increasing post-ischemic reperfusion time (Fig.S2) and calpain inhibition by calpeptin significantly reduced MP production in the present experiments [31]. Whether agents to sequester mobilisation of ionic calcium or calpain antagonists could have therapeutic application in hepatic IRI requires further study. Our previously ultrastructural studies revealed extensive blebbing of the sinusoidal endothelium at 30 min post-ischemic reperfusion [10]. The present results show this is the origin of the MPs that circulate within 15 min of hepatic IRI. This potentially does have therapeutic implications for early intervention in IRI. Thus, we addressed the suggestion of Albano [17] that Diannexin, by binding to PS, might inhibit MP release, thereby interfering with the inflammatory and procoagulant effects normally executed by MPs. This was shown unambiguously to be the case. Moreover, residual MPs from Diannexin pre-treated mice displayed less pro-inflammatory and pro-oxidant potential and activity. Lipid rafts of the cell membrane may also play a role in MP release [32]; annexin V is known to bind to lipid rafts of cells and to PS with high affinity [33]. It is therefore also plausible that Diannexin, by binding to lipid rafts, prevents the distortion of the plasma membrane surface that is a pre-requisite for vesicle-budding to form MPs.

In summary, our data indicate that MPs are released during liver IRI and that these MPs are pro-inflammatory, platelet activating, and directly pathogenic to hepatocytes *in vitro*. By simulation experiments, we have shown that MP production, at least in hepatocytes, is modulated by oxidative stress, TNF and calcium fluxes, known pathogenic factors in IR. Not all MPs are the same; by selective study of cell-derived MP subfractions, we showed those from KCs/macrophages and platelets may be particularly pro-inflammatory and particularly injurious to hepatocytes. Whether this is due to higher AA and F2-isoprostane content requires further investigation. Meanwhile, the clear finding that Diannexin protects against MP production and MP-induced injury has therapeutic implications. Thus, we have already shown that Diannexin exerts both preventive and therapeutic effects against hepatic IRI, the latter up to 24 hrs after post-ischemic liver reperfusion [10]. Taken together, the present results reveal pivotal pathogenic mechanisms that *initiate* and likely *perpetuate* hepatic IRI during liver surgery and transplantation. Implication of MP formation should facilitate the development of clinically applicable and effective strategies against IRI.

## Supporting Information

Figure S1 ***N*** **-acetylcysteine (NAC) and Diannexin are hepatoprotective against liver ischemia reperfusion injury (IRI) by reducing oxidative stress potentials of MPs** ***in vitro*** **and** ***in vivo*** **.** **A**. Serum ALT release in mice pre-treated with NAC (150 mg/kg ip) 15 min prior to 60 min ischemia and 24 hr reperfusion (n = 5 mice per experimental cohort). *p<0.05 NAC-treated mice vs. naïve. #p<0.05 naïve vs. sham or NAC alone. **B**. NAC pre-treatment significantly reduces generation of MPs post-IRI. *p<0.05 NAC-treated mice vs. naïve. #p<0.05 naïve vs. sham or NAC alone. **C**. NAC rescues hepatic GSH levels and significantly decreases glutathione oxidation levels (oxidized glutathione/reduced glutathione, GSSG/GSH ratio) compared to naïve mice. Diannexin also restores hepatic GSH levels and significantly decreases glutathione oxidation levels compared to naïve mice. MPs were obtained from mice pre-treated with 1 mg/kg iv Diannexin 5 min prior to 60 min liver IR and results compared with vehicle-treated/naïve mice subject to IR (n = 5 mice per experimental cohort). *p<0.05 NAC- or Diannexin-treated mice vs. naïve. #p<0.05 NAC- or Diannexin-treated vs. sham (individual agents alone). &§p<0.05 vs. all experimental groups. **D**. MPs derived from mice subjected to 60 min ischemia and 2 hr reperfusion, were incubated with primary murine hepatocytes pre-treated with NAC (vs. naïve) for 24 hr. MPs significantly increased glutathione oxidation (GSSG/GSH) in primary hepatocytes which was significantly inhibited by NAC. Data presented as mean ± SD (n  = 5 mice per group, glutathione experiments done in triplicate thus n = 15). *p<0.05 NAC-treated mice vs. naïve or vehicle (PBS, phosphate buffered saline). #p<0.05 NAC-treated vs. naïve mice. &p<0.05 vs. all experimental groups.
(EPS)

Figure S2 **Hepatic calpain activity escalates with increasing reperfusion time in mice subjected to 60 min ischemia (Fluorogenic Calpain Activity kit, QIA120, Calbiochem, Germany).** * p<0.05 indicated experimental groups vs. sham. # p<0.05 2 and 24 hr reperfusion vs. 15 min reperfusion. + p<0.05 24 hr reperfusion vs. 2 hr reperfusion. & p<0.05 24 hr reperfusion vs. sham, 15 min, 30 min, 2 hr reperfusion.
(EPS)

Table S1 A. Table listing CD markers used, their cell-specificities and where they were sourced from (company, city, state in the USA). B. Table listing CD markers used, their working dilutions, amount utilised in experiments and respective isotype controls.
(DOC)

Materials and Methods S1 **Supplementary Materials and Methods.**
(DOCX)

References S1 **Supplementary References.**
(DOCX)
