# Supplementary material for: Microparticles Mediate Hepatic Ischemia-Reperfusion Injury and Are the Targets of Diannexin (ASP8597)
Source: PLoS One. 2014 Sep 15;9(9):e104376. doi: 10.1371/journal.pone.0104376 (PMC4164362; doi:10.1371/journal.pone.0104376)
Supplement: Table S1 — A. Table listing CD markers used, their cell-specificities and where they were sourced from (company, city, state in the USA). B. Table listing CD markers used, their working dilutions, amount utilised in experiments and respective isotype controls. (DOC) [file pone.0104376.s003.doc]

**SUPPLEMENTARY TABLE**

**Supplementary Table 1**

1. **Table listing CD markers used, their cell-specificities and where they were sourced from (company, city, state in the USA)**

| **CD markers** | **Cell-specificity** | **Source** |
| --- | --- | --- |
| CD41-FITC | platelets | Biolegend, San Diego, CA |
| CD-62P-FITCE (P-selectin) | activated platelets | Biolegend, San Diego, CA |
| F4/80-PE | macrophages | Biolegend, San Diego, CA |
| CD144-PE (VE-cadherin) | endothelial cells | Biolegend, San Diego, CA |
| Ly-6G-PerCp | leukocytes | Biolegend, San Diego, CA |
| CD8-FITC | cytotoxic T cells | Biolegend, San Diego, CA |
| CD3-PerCp | T cells | Biolegend, San Diego, CA |

1. **Table listing CD markers used, their working dilutions, amount utilised in experiments and respective isotype controls**

| **CD marker** | **Working dilution** | **Amount of Antibody** | **Isotype control** |
| --- | --- | --- | --- |
| CD144 | 1:200 | 0.1 μg | PE Rat IgG1 |
| CD41 | 1:200 | 0.25 μg | FITC Rat IgG1 |
| CD62P | 1:200 | 0.25 μg | FITC Mouse IgG1 |
| F4/80 | 1:200 | 0.1 μg | PE Rat IgG2a |
| Ly6G | 1:200 | 0.1 μg | PerCP/Cy5.5 Rat IgG2a |
