# Supplementary material for: Microparticles Mediate Hepatic Ischemia-Reperfusion Injury and Are the Targets of Diannexin (ASP8597)
Source: PLoS One. 2014 Sep 15;9(9):e104376. doi: 10.1371/journal.pone.0104376 (PMC4164362; doi:10.1371/journal.pone.0104376)
Supplement: Materials and Methods S1 — Supplementary Materials and Methods. (DOCX) [file pone.0104376.s004.docx]

**Supplementary Materials and Methods**

**Microparticle (MP) isolation protocol**

Blood was collected by cardiac puncture, spun for 15min (1,500g) and plasma re-centrifuged 2min (13,000g). MPs were sedimented from plasma by prolonged centrifugation at 100,000 *g* for 60 min. MPs were recovered from primary hepatocyte culture medium using a similar protocol. Thereafter, MP pellets were resuspended in phosphate-buffered saline (PBS) before FACS analysis. Plasma containing MPs may also be stored at -80°C and recovered for later analyses by ultra-centrifugation (100,000*g* for 60min). We measured MP size using nano-fluorescent polystyrene particle beads and FACS (Spherotech, IL). The gating strategy employed was based initially on size of MPs, 0.1 – 1.0um and thereafter, analyses of subpopulations were carried out using cell-derived markers/’tags’(Supplementary Table 1).

Blood collection via cardiac puncture in citrated tubes

↓

Centrifugation at 1,500 *g* at 21°C for 15 min

↓

Platelet-rich plasma (PRP) centrifuged at 13,000 *g* for 2 min

↓

Platelet-free plasma (PFP) ultracentrifuged at 100,000 *g* for 60 min

↓

Pellets resuspended in sterile phosphate-buffered saline (PBS)

↓

FACS sorting using cell-derived markers

**Microparticles and fusion events**

Fusion of MPs is known to occur *in vitro* as well as *in vivo (supplementary refs to methods i, ii).* For this reason, each MP can conceivably carry multiple markers from different cells of origin. The gating strategy we employed was based initially on size of MPs, 0.1 – 1.0μm with the aid of nanofluorescent particles (Spherotech, Libertyville, IL). Thereafter, analyses of subpopulations were carried out using cell-derived markers/’tags’. Hence, the sum of all the percentages derived by FACS for each cell subset can feasibly exceed 100%, as shown in Fig 1C.

- 1. Palmisano G, et al (2012) Characterization of membrane-shed microvesicles from cytokine-stimulated beta-cells using proteomic strategies. Mol Cell Proteomics 11:230-243.
  2. Faille D, et al (2012) Endocytosis and intracellular processing of platelet microparticles by brain endothelial cells. J Cell Mol Med 16:1731-1738.

**MP detection by ELISA**

MP production was determined by a commercial assay which quantifies phosphatidylserine (PS) production (Zymuphen MP Activity, HYPHEN BioMed, France). In this assay, PS^+^ MPs are captured on an ELISA plate coated with annexin FV-streptavidin and incubated with Factor Va, Xa and prothrombin to form the prothrombinase complex which cleaves prothrombin to thrombin. After addition of thrombin-specific chromogenic substrate and 2% citric acid, absorbance was measured at 405nm. Values are expressed as PS (and thus MP) equivalents.

**Western blot analysis of ASGPR, ICAM-1, VCAM, E-selectin, P-selectin, VE-cadherin, IκB-α, p65, COX-2, PKC-δ, JNK1/2, phosphorylated-JNK and measurement of serum TNF**

MP pellets re-suspended in platelet-free plasma were subjected to protein estimation (Bradford assay)*.* 20μg of MP protein was resolved by 12% SDS-PAGE under reducing conditions, transferred to PVDF membranes (Millipore, Billerica, MA, and probed for ASGPR, ICAM-1, VCAM, E-selectin, P-selectin, IκB-α, COX-2, PKC−δ, JNK1/2, phosphorylated-JNK (all Santa Cruz Biotechnology, CA) and VE-cadherin (BD Biosciences). Protein expression levels were quantified by densitometry using Fujifilm LAS-4000_mini_ imaging software. Serum TNF concentrations were measured by ELISA (R & D Systems, Minneapolis, MN).

**Measurement of mitochondrial membrane potential and oxidative stress**

Tetramethylrhodamine methyl ester (TMRM)(Sigma, St Louis, MO), a fluorescent dye retained in normal mitochondria, was used as marker of mitochondrial membrane potential (ΔΨ_m_). Hepatocytes were incubated in media containing 50nM TMRM for 15min at 37°C (23). Cells were then washed 3 times with PBS and TMRM was imaged using Olympus IX71 fluorescence microscope. Intracellular oxidative stress levels were measured by 2,7-dichlororofluorescein diacetate (DCFDA, Sigma, St Louis, MO) fluorescence. In the dark, murine primary hepatocytes were incubated 1 µM DCFH2-DA for 30 min at 37°C, and washed prior to imaging. Red fluorescence (TMRM) was excited at 543 or 568 nm, and emission imaged at > 590 nm; green fluorescence (DCFDA) was excited at 488 nm, and emission was collected through a 515-nm to 560-nm band pass filter.

**Serum hyaluronic acid (HA) ELISA**

HA measurements were determined by ELISA (catalogue #K-1200, Echelon Biosciences, Salt Lake City, UT).

***N*-acetylcysteine (NAC) pre-treatment in mice**

Animal protocols were approved by the Australian National University’s Animal Ethics Committee. Groups (n=5) of male C57BL6 mice age 8-12 weeks were anesthetized (ketamine 100 mg/kg, xylazine 20mg/kg intraperitoneally, ip) and subjected to partial hepatic ischemia. Where indicated, mice were treated with a single and clinically relevant dose of *N*-acetylcysteine (150 mg/kg ip, Sigma, *supplementary references to methods iii, iv*) 15min before liver IRI.

- 1. Teoh N, Chitturi S, Farrell G (2006) Chpt 83: Liver Diseases Caused by Drugs. *Sleisenger and Fordtran’s Textbook of Gastrointestinal Disease*. 8^th^ Edition. Eds. Feldman M, Friedman L.S., Brandt L.J. Philadelphia, Elsevier.
  2. Sener G, et al (2003) Melatonin and N-acetylcysteine have beneficial effects during hepatic ischemia and reperfusion. Life Sci 72:2707-18

**Reduced glutathione (GSH) and oxidized glutathione (GSSG) studies**

GSH and GSSG was determined using a kit (catalogue #703002 Cayman Chemicals, Ann Arbor, MI) in murine primary hepatocytes and whole liver lysates obtained from mice subjected to 60min ischemia and 24hr reperfusion.
