# Supplementary material for: Microparticles Mediate Hepatic Ischemia-Reperfusion Injury and Are the Targets of Diannexin (ASP8597)
Source: PLoS One. 2014 Sep 15;9(9):e104376. doi: 10.1371/journal.pone.0104376 (PMC4164362; doi:10.1371/journal.pone.0104376)
Supplement: References S1 — Supplementary References. (DOCX) [file pone.0104376.s005.docx]

**Supplementary References to Manuscript**

S20. Tsai IJ, et al (2009) 20-HETE and F2-isoprostanes in the metabolic syndrome. Free Rad Biology Med 46:263-270.

S21. Proudfoot JM, Barden AE, Loke WM, Croft KD, Mori TA (2009) HDL is the major lipoprotein carrier of plasma F2-isoprostanes. J Lipid Research 50:716-722.

S22. Jy W, Horstman LL, Jimenez JJ, Ahn YS (2004) Measuring circulating cell-derived microparticles. J Thromb Hemostasis 2:1842-1851.

S23. **Han MS, Park SY**, et al (2008) Lysophosphatidylcholine as a death effector in the lipoapoptosis of hepatocytes. J Lipid Research 49:84-97.

S24. **Fourcade O, Marie-Francoise S**, et al (1995) Secretory phospholipase A2 generates the novel lipid mediator lysophosphatidic acid in membrane microvesicles shed from activated cells. Cell 80:919-927.

S25. Barry OP, Pratico D, Lawson JA, Fitzgerald GA (1997) Transcellular activation of platelets and endothelial cells by bioactive lipids in platelet microparticles. J Clin Invest 99:2118 -2127.

S26. van Velzen JF, Laros-van Gorkom BA, Pop GA, van Heerde WL (2012) Multicolor flow cytometry for evaluation of platelet surface antigens and activation markers. Thromb Res 130:92-98.

S27. Heuser JE, Anderson, RG (1989) Hypertonic media inhibit receptor mediated endocytosis by blocking clathrin-coated pit formation. J Cell Biol 108:389–400.

S28. Pasquet JM, Dachary-Pringent J, Nurden AT (1996) Calcium influx is a determining factor of calpain activation and microparticle formation in platelets. Eur J Biochem 239:647-654.

S29. Combes V, Coltel N, Faille D, Wassmer SC, Grau GE (2006) Cerebral malaria: role of microparticles and platelets in alterations of the blood-brain barrier. Int J Parasitol 36:541-456.

S30. Uehara T, et al. JNK mediates hepatic ischemia reperfusion injury (2005) J Hepatol 42:850-859.

S31. Yano Y, et al (1993) The effects of calpeptin on agonist induced microparticle formation from the platelet plasma membrane. Thromb Res 71:385-96.

S32. Dillon SR, Mancini M, Rosen A, Schlissel MS (2000) Annexin V binds to viable B cells and colocalizes with a marker of lipid rafts upon B cell receptor activation. J Immunol 164:1322-1332.

S33. Rand ML, et al (2012) Diannexin, an annexin A5 homodimer, binds phosphatidylserine with high affinity and is a potent inhibitor of platelet-mediated events during thrombus formation. J Thromb Haemost 10:1109-1119.

**Authors names in bold designate shared co-first authorship**
